# Iatrogenic early onset cerebral amyloid angiopathy 30 years after cerebral trauma with neurosurgery: vascular amyloid deposits are made up of both Aβ40 and Aβ42

**DOI:** 10.1186/s40478-019-0719-1

**Published:** 2019-05-02

**Authors:** Giorgio Giaccone, Emanuela Maderna, Gianluca Marucci, Marcella Catania, Alessandra Erbetta, Luisa Chiapparini, Antonio Indaco, Paola Caroppo, Anna Bersano, Eugenio Parati, Giuseppe Di Fede, Luigi Caputi

**Affiliations:** 1 Fondazione IRCCS Istituto Neurologico Carlo Besta, Unit of Neurology 5 – Neuropathology, Milan, Italy; 2 Fondazione IRCCS Istituto Neurologico Carlo Besta, Unit of Neurology 9 - Cerebrovascular Diseases, Milan, Italy; 3 Fondazione IRCCS Istituto Neurologico Carlo Besta, Unit of Neuroradiology, Milan, Italy

Aβ protein is the main component of the amyloid of Alzheimer disease (AD) that builds up extracellularly in the neuropil as senile plaques (SP) and in the vessel walls as cerebral amyloid angiopathy (CAA) [14]. In some cases, CAA dominates pathologically over SP and this corresponds to a clinical presentation with multiple cerebral hemorrhages rather than degenerative dementia. The former can either present as a sporadic disease or as an hereditary condition (cerebral hemorrhage with amyloidosis, HCHWA) associated with specific *APP* mutations [3, 13].

Although the experimental seeding of Aβ is a well-known phenomenon [11], examples of Aβ transmission in humans have not been reported until recently. Amyloid Aβ transmission by a prion-like mechanism has been postulated at first on the basis of the neuropathological findings in patients with iatrogenic Creutzfeldt-Jakob disease [4, 12] and later in young adult individuals with early onset CAA who had a history of neurosurgery or other invasive medical procedures [2, 7–9]. In these papers, young adults (aged 30 to 57) have been reported in whom pathologically proven CAA has been linked with intrusive medical procedures performed some decades before consisting in neurosurgery with or without dura mater grafting and embolization of external carotid artery by dural extracts.

Here we report a 29-year old man who abruptly presented a thunderclap headache, associated with bilateral blurred vision. Left homonymous hemianopia was present and CT scan showed an acute hemorrhage in the right parietal and occipital lobes with perilesional edema. 5 months later, a similar episode occurred and neuroimaging showed an acute hemorrhage in the left parietal and occipital lobes with perilesion edema.

After other 5 months, the patient had acute headache and left facio-brachio-crural weakness. Brain CT and MRI showed an hemorrhage in the right frontal and parietal lobes with perilesional edema, with slow radiological improvement. 1 month later, bilateral worsening of the visual acuity was observed, due to an acute hemorrhage in the left parietal and occipital lobes. A further increase of the volume of the hemorrhage associated with headache was observed 30 days later.

3 months after this episode, he underwent neurosurgery with open left temporo-occipital meningeal and cerebral biopsy.

**Fig. 1:**
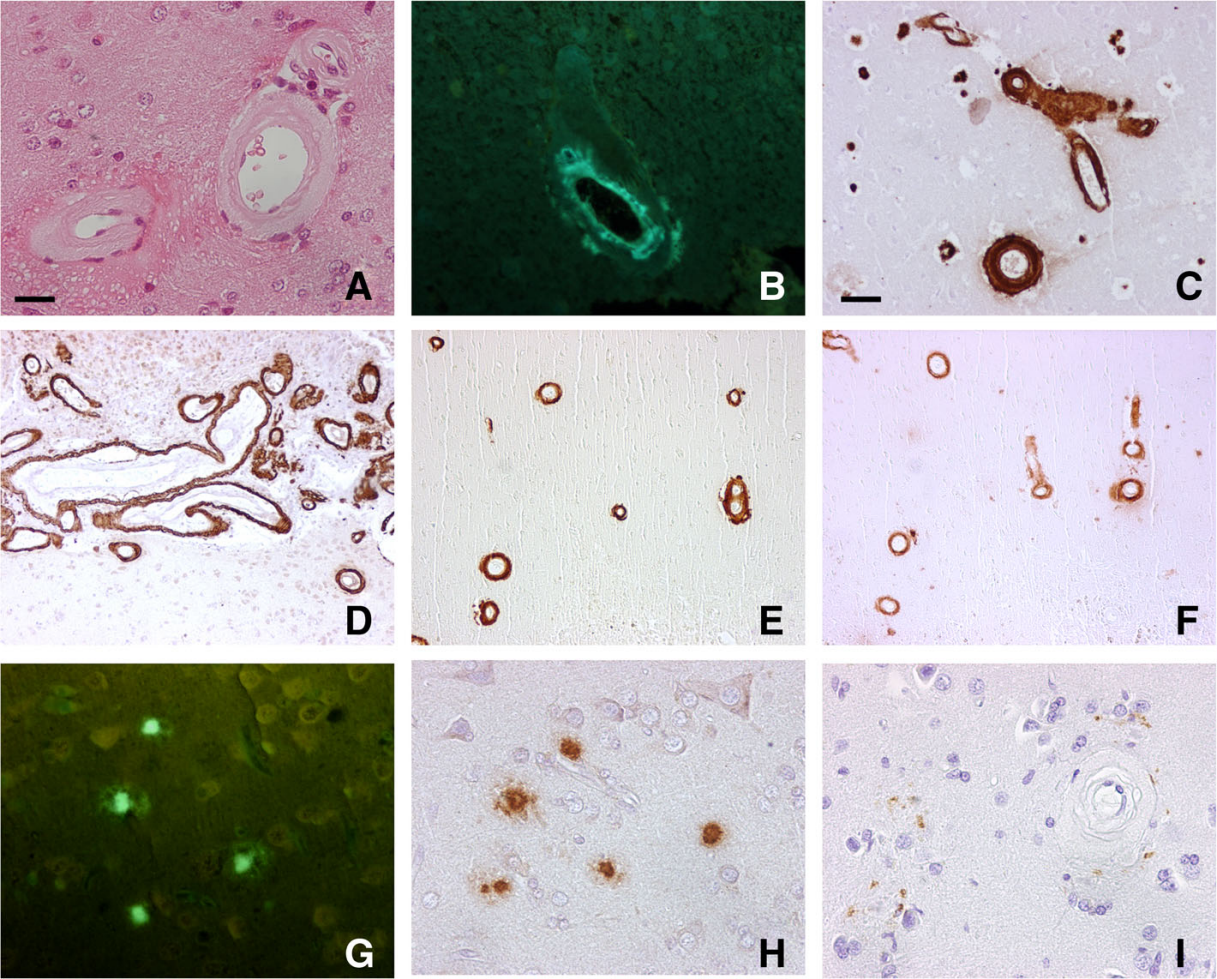
Neuropathologic findings of the cerebral biopsy. Severe amyloid angiopathy appeared as thickening of the wall of parenchymal arterioles (**a**, Haematoxylin &Eosin) where amorphous material, fluorescent after thioflavine S treatment, built up (**b**, thioflavine S). When antibody 4G8 was used (mouse monoclonal, 1:2000, after 80% formic acid for 20 min) that recognizes the different Aβ species (epitope at residues 17–24 of Aβ), immunoreactivity was intense both in parenchymal (**c**) and leptomeningeal vessels (**d**). Both the antibody specific for Aβ40 (mouse monoclonal, Covance, 1:1000, after 80% formic acid for 20 min)(**e**) and that specific for Aβ42 (mouse monoclonal, Covance, 1:500, after 80% formic acid for 20 min)(**f**) strongly decorated the amyloid-laden vessels. Compact Aβ deposits were present in the neuropil (**g**, thioflavine S) and were intensely immunolabeled by anti-Aβ42 (not shown) and 4G8 (**h**), while tau pathology was minimal, appearing as cellular profiles immunopositive for anti-phosphorylated tau antibody AT8 (mouse monoclonal, Biosource, 1:300) often surrounding amyloid laden vessels (**i**). Immunolabeling was visualized by the Envision Plus/Horseradish Peroxidase System (DakoCytomation) using 3–3′-diaminobenzidine (brown reaction product) as chromogen. Bar in A = 25 μm (A,B,G,H and I are the same magnification); bar in C = 100 μm (C,D,E and F are the same magnification)

The neuropathological examination revealed severe CAA in many leptomeningeal and cortical vessels, (Fig. 1a-d). Immunohistochemistry for Aβ showed also the presence of a moderate number of compact senile plaques while neurofibrillary tangles were absent and tau pathology was minimal appearing as isolated neuronal processes immunoreactive for phosphorylated tau (Fig. 1g-i). The immunostaining with specific antibodies disclosed that both Aβ40 and Aβ42 were consistently represented in the vascular amyloid deposits (Fig. 1e,f).

CSF analyses revealed slightly low Aβ42 (497 pg/mL, normal value > 500), normal total tau (207 pg/mL, normal value < 500) and phospho-tau P181 (41 pg/mL, normal value < 61). Genetic testing excluded known mutations involved in hereditary Aβ-CAA (APP, PSEN1; PSEN2). The APOE genotype was ε3/ ε3. No family history for neurological diseases was reported.

**Fig. 2:**
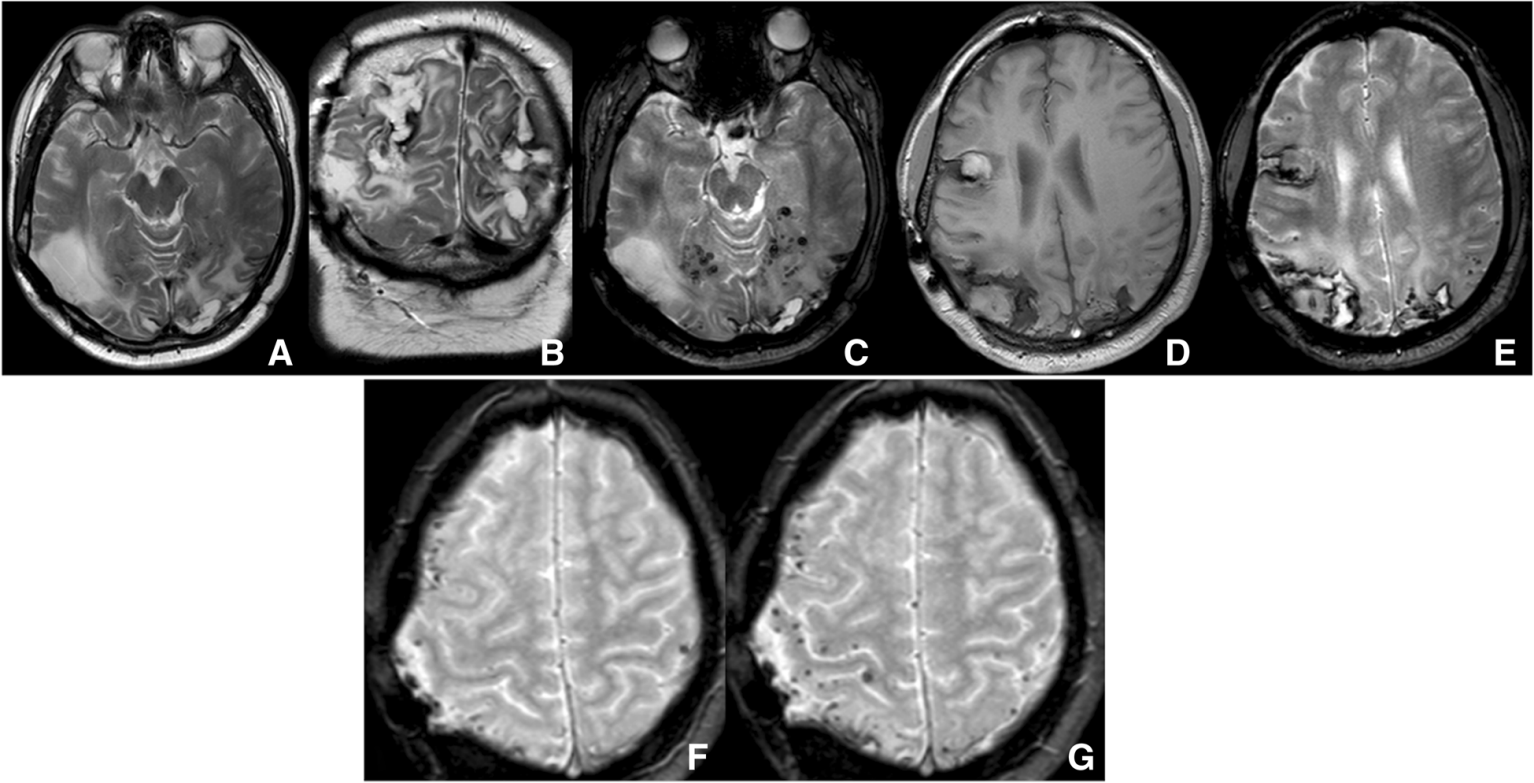
MR findings. MR examination obtained on a 3Tesla unit (**a-e**), and 1.5Tesla unit (**f, g**). Axial (**a**) and coronal (B) T2 weighted images show the outcome of long-standing surgery with cranioplasty in the right temporo-parietal region and multiple, posterior cortical and subcortical lesions with bleeding areas and hemosiderin. Axial T2* gradient-echo (**c**) image shows multiple tiny scattered hypointensities consistent with microhemmorhages in posterior regions. In T1 wi (**d**) a recent right frontal hematoma is shown. Leptomeningeal microhemorrhages are disseminated also in the frontal and parietal lobes as demonstrated in T2*gradient-echo image (**e**). Note in T2*gradient-echo images (**f** and **g**), the progression of punctate microhemorrhages in 4-years follow-up, evident also in 1,5 Tesla

Serial brain MRI before and after brain biopsy demonstrated lobar hemorrhages and diffuse cortical-subcortical micro-hemorrhages with progression during follow-up (Fig. 2). Since brain biopsy, about three episodes per week of brief aphasia and long-lasting (one hour) drowsiness associated with further blurred vision occurred. Hypertension was not reported before the first hemorrhage, but it developed 1 year after requiring pharmacologic treatment (beta-blocker, ACE inhibitor and diuretic). Cognitive impairment was not present neither before the recurrent cerebral hemorrhages nor at follow up.

At the age of one year, the patient had a traumatic brain injury due to car crash. CT scan revealed the swelling in the right frontal-temporal-parietal and occipital lobes with underneath bone fracture. Three months later he underwent a neurosurgical procedure for the unstable bone fracture through reconstruction of the bone borders and the dura mater (Bologna, Italy, December 1986). 20 years later the patient underwent cranioplasty (Milano, Italy, January 2007). No further details on the procedures were available nor data allowing to confirm or exclude the use of cadaveric dura mater graft. Even if a definite number is not available, the use of cadaveric dura graft has been widely utilized in Italy until the late 1980s. It is also noteworthy that the neurosurgical departments where the patient underwent the two reconstructive procedures are not pediatric neurosurgical units and therefore the same sets of instruments used for adult neurosurgery could have also been used for our patient

As for the similar cases recently reported [2, 7–9] the likelihood that the traumatic injury and neurosurgery in infancy is causative of early onset CAA in our patient is very high: the very young age (first cerebral hemorrhage at 29 years, the youngest of the patients reported until now with this syndrome), the absence of mutations in genes associated with early Aβ pathology, the severity of CAA, are all significant elements supporting this hypothesis.

Jaunmuktane et al. described 4 patients with CAA aged 31–57 who underwent neurosurgical procedure decades earlier for trauma, cerebral tumor, congenital malformation and syringomyelia, without confirmatory evidence of dural grafts, and concluded for a possible transmission by surgical instruments carrying traces of misfolded Aβ protein.

Hervè et al. (1 patient, aged 46) and Banerjee et al. (3 patients, aged 34–48) reported a similar picture of iatrogenic early onset-CAA in patients with previous documented exposure to dural graft (3 patients) and to arterial embolization by dural extracts (1 patient), pointing to contaminated dura as the source of misfolded Aβ protein.

Therefore, the mechanisms by which transmission of Aβ pathology may occur are not fully established: contaminated neurosurgical instruments or exposure to dura mater (by grafting or embolization) containing Aβ seeds are the main suspect. It is noteworthy that Aβ traces have been detected in dura mater [10]. Another possibility is that the brain trauma (either external insults affecting the head or that secondary to neurosurgery) caused the disturbance of clearing system of cerebral Aβ, such as glymphatic system and/or intramural periarterial drainage pathways [7].

Likewise, unexplained is why in these iatrogenic cases Aβ preferentially accumulates in the walls of the cerebral vessels rather than in brain parenchyma even if it consists of both Aβ42 and Aβ40 species. This finding, if confirmed in other iatrogenic CAA patients, may be relevant, since both in sporadic CAA patients and genetic HCHWA in large vessel CAA Aβ40 affects vascular walls more frequently and more severely than Aβ42 [1, 5, 6].

Our report increases the number and the data available about patients with early-onset iatrogenic Aβ-CAA. It may be postulated that similar patients exist in older age range, but are difficult to identify as the occurrence of CAA becomes less unusual with advancing age. In any cases, asking about a prior history of neurosurgery should become mandatory in patients with CAA of any age.
